# ﻿Description of a male *Zimirina corsica* sp. nov. (Arachnida, Araneae) – the first Prodidomidae species reported on the European territory of France

**DOI:** 10.3897/zookeys.1250.147215

**Published:** 2025-09-01

**Authors:** Alain Canard, Pierre Devogel, Pierre Oger, Christine Rollard, Olivier Villepoux, Frédéric Ysnel

**Affiliations:** 1 Université de Rennes-MNHN – UMR, 8067 BOREA, Campus de Beaulieu, 35042, Rennes Cedex, France Université de Rennes-MNHN – UMR Rennes Cedex France; 2 Groupe d’Etude des Invertébrés Armoricains–Campus de Beaulieu, 35042, Rennes Cedex, France Groupe d’Etude des Invertébrés Armoricains–Campus de Beaulieu Rennes Cedex France; 3 Rue du Grand Vivier 14, B-4217 Waret l’Évêque, Belgium Unaffiliated Waret l’Évêque Belgium; 4 Muséum national d’histoire naturelle, Département Origines & Evolution, ISYEB, UMR 7205 CNRS MNHN SU EPHE UA, CP 53, 61 rue Buffon, 75005, Paris, France Muséum national d’histoire naturelle Paris France; 5 8, rue des Pérailles, 43100 Paulhac, France Unaffiliated Paulhac France

**Keywords:** French Mediterranean island, long-spinneret ground spiders, new species, taxonomy, *
Zimirina
*

## Abstract

A new Prodidomidae species (genus *Zimirina* Dalmas) is proposed based on the description of a male specimen caught in Corsica. The specimen can be distinguished from other *Zimirina* males by the shape of its tegulum and cymbium. Considering that it differs in a number of characters from all other male *Zimirina* we propose to name this new species *Zimirina corsica***sp. nov.**, referring to the type locality.

## ﻿Introduction

Prodidomidae species were initially grouped by Simon (1884) as « un petit groupe très naturel dans la famille des Drassidae » then individualized as a family from Simon (1893) to Platnick (1990). Phylogenetic studies provisionally called into question the existence of this family, and Prodidomidae were transiently integrated among Gnaphosidae (Azevedo et al. 2018; Rodrigues and Rheims 2020); however, the most recent study (Azevedo et al. 2022) raised them back to the family level. They are characterized by very original, clearly disjointed anterior spinnerets, the anterior distant from the posterior, each equipped with particularly long piriform gland spigots with greatly elongated bases bearing long plumose setae, and by their mutic chelicerae (neither points, nor spines, nor teeth), often divergent with long, thin fangs (de Dalmas 1919; Cooke 1964; Dippenaar-Schoeman and Jocqué 1997; Wunderlich J 2011). The family contains 195 species grouped into 24 genera, and is mainly found in warm regions around the world. Very few individuals of this family have been reported in the Western Palearctic region, in particular in European countries.

The habitat of the species is variable: some live under stones or in underground cavities (de Dalmas 1919; Cooke 1964) while others are synanthropic. Until now, the biology of many species in this family has hardly been studied due to their small size at the adult stage.

Among Prodidomidae, the genus *Zimirina* Dalmas, 1919 is mainly distinguished from the other Palearctic genera *Prodidomus* Hentsz, 1847 and *Zimiris* Simon, 1882 by 1) the spigots of their anterior spinnerets longer than the basal article, 2) the absence of a fovea (present in *Zimiris)*, and 3) non-enlarged spinnerets, unlike *Prodidomus* (de Dalmas 1919; Cooke 1964). Fifteen *Zimirina* species are currently identified, 13 of which have been found in the Western Palearctic region (World Spider Catalog 2025). Most of them are known from Macaronesia: seven taxa are described from the Canaries, and one from Madeira (Table 1). The biology of *Zimirina* species is little known, and the rarity of that genus is particularly exceptional among spiders. Like most Prodidomidae, *Zimirina* species are known to colonize thermophilic environments with pioneer vegetation, e.g., mining landfills (Pantini et al. 2013) or arid scree. They can move very quickly over short distances, interspersed with periods of slow movements (Crespo et al. 2013) (rather like *Oonops domesticus* Dalmas, 1916), which makes them difficult to spot on the ground.

## ﻿Material and methods

The specimen was examined and measured using an Olympus SZ binocular lens equipped with a Moticam camera for taking photographs. Its body length was measured from the anterior margin of its clypeus to the posterior end of its abdomen, spinnerets excluded. The male copulatory bulb was examined, and the specimen was stored in 75% ethanol. It was described from four aspects: dorsal, ventral, prolateral and retrolateral. The type was deposited in the University Museum of Rennes.

## ﻿Results

### ﻿ Zimirina corsica

Taxon classificationAnimaliaAraneaeProdidomidae

Canard & Ysnel sp. nov.

615684EA-2057-5DCB-9104-3CBF9C7C13AA

https://zoobank.org/B731C0B1-6324-45D2-B89B-13878326BECA

Figs 10, 13, 14–16

#### Type locality.

Corsica (European territory of France).

#### Etymology.

The specific name is a noun in apposition referring to the type locality.

#### Material examined.

***Holotype*** • 1 male, France, Haute-Corse, Omessa (24/5/2018) (42°21'13"N, 2°11'56"E, altitude 464 m, May 24, 2018), house wall (Mus. Rennes, n° 2018-0169).

#### Diagnosis.

**Figures 1–13.**
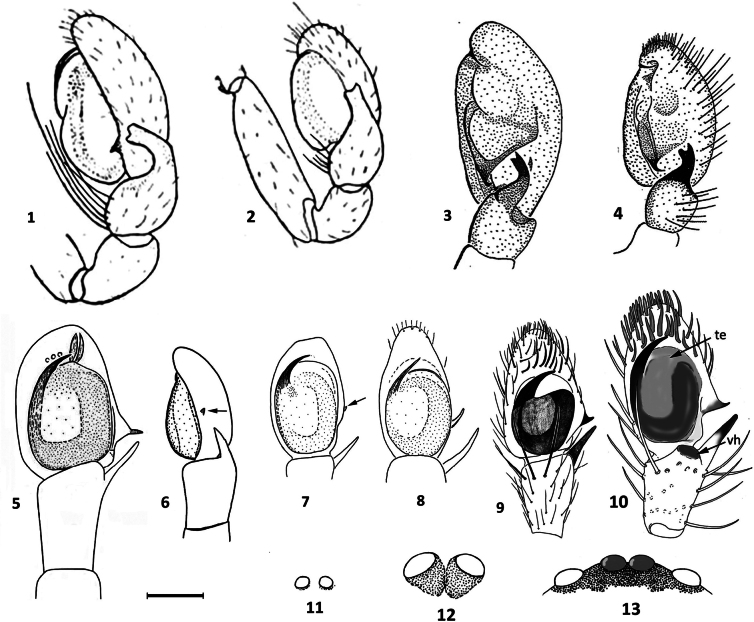
Lateral views of palps of known males of European and North African species. **1, 2.** (According to de Dalmas 1919): **1.***Z. deserticola*; **2.***Z. penicillata*; **3, 4.** (According to Cooke 1964): **3.***Z. tenuidens*; **4.***Z. hirsuta*; **5–8.** (Adapted from Wunderlich 1987, 1992, 2011): **5.***Z. spinicymbia*; **6.***Z. cineris*; **7.***Z. gomerae*; **8.***Z. nabavii*; **9.***Z. lepida* (according to Crespo et al. 2013); **10.***Z. corsica* sp. nov.; **11, 12.** Anterior median eyes (according to Wunderlich 2011): **11.***Z. nabavii*; **12.***Z. cineris*; **13.** anterior eye line in *Z. corsica* sp. nov. Abbreviations: te, tegulum; vh, ventral hump. Scale bar: 0.1 mm.

*Zimirina corsica* sp. nov. can be distinguished from the males of other *Zimirina* species by the following characters: the palp of the North African species, *Z. deserticola* Dalmas, 1919, *Z. penicillata* (Simon, 1893), *Z. tenuidens* Denis, 1956, and the Macaronesian species *Z. hirsuta* Cooke, 1964, have a short tibia and a RTA with a bifid end (Figs 1–4); the palp of *Z. spinicymbia* Wunderlich, 1992 has a longer tibia than the cymbium (Fig. 5); the cymbial process is very small in *Z. cineris* Cooke, 1964 and in *Z. gomerae* (Schmidt, 1981) (Figs 6, 7); the anterior median eyes in *Z. nabavii* Wunderlich, 2011 are much smaller than the lateral ones (Fig. 11); the tegulum does not fill the entire alveolus in *Z. lepida* (Blackwall, 1859), there is no ventral hump on the tibia (Fig. 9), and the apical setae of the cymbium bristles do not show a truncated appearance as in *Z. corsica* sp. nov.

#### Description.

Total length: 3.1 mm; length of the cephalothorax: 1.18 mm, width: 0.93; length of the sternum: 0.82 mm, width: 0.58 mm. Leg formula 4123: PI: 3.53 mm PII: 2.72 mm; PIII: 2.64 mm; PIV: 4.2 mm.

**Figures 14–16.**
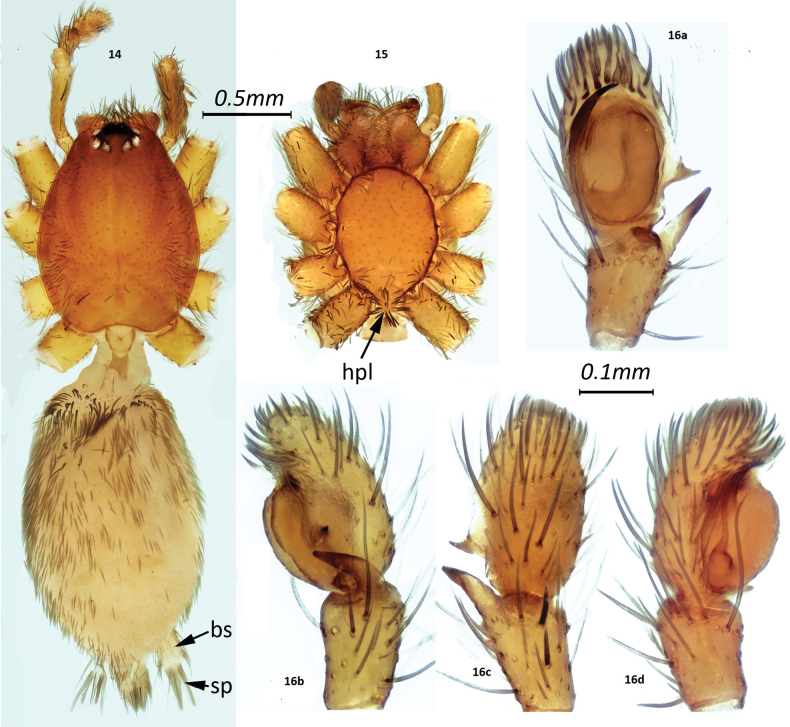
*Zimirina corsica* sp. nov. **14.** Habitus of male; **15.** Sternum; **16a–d.** Male palp: **a.** Ventral; **b.** Retrolateral; **c.** Dorsal; **d.** Prolateral. Abbreviations: bs, spinneret basal segment; sp, spigots; hpl, hairy posterior lobe.

Cephalothorax provided on margin with fairly thick setae, aligned (Fig. 14), with posterior notch, without thoracic fovea (note: many setae have fallen off). Eyes are arranged in a circle, with anterior row recurved, posterior row procurved. Eye group approximately 1.7 times wider than long (0.28 mm/0.17 mm). Eyes of approximately equal size are arranged in an open circle. Posterior median eyes are separated from each other by approximately one diameter. Sternum continuous oval, without marked angles at the insertion of the legs and provided with tuft of radiating setae at posterior end (Fig. 15). Integument pale testaceous with cephalothorax slightly darker. Legs entirely pale testaceous.

***Pedipalp*.** The end of the tibia has a thin apical blunt process, curved forward in retrolateral view (Figs 10, 16a). There is a dark ventral bump at the base of this spine. Cymbium with a retrolateral dentiform spur is located in the basal third; it is finished with a fairly straight brush of bristles (Fig. 16c, d). Tegulum occupies the entire alveolus. Embolus originates at 9 o’clock position and curved prolaterally to 12 o’clock position. Palpus femur (0.43 mm) is approximately three times as long as the tibia (0.15). Cymbium is less than twice as long as the tibia (1.7 times).

**Female.** Unknown.

## ﻿Discussion

**Figure 17.**
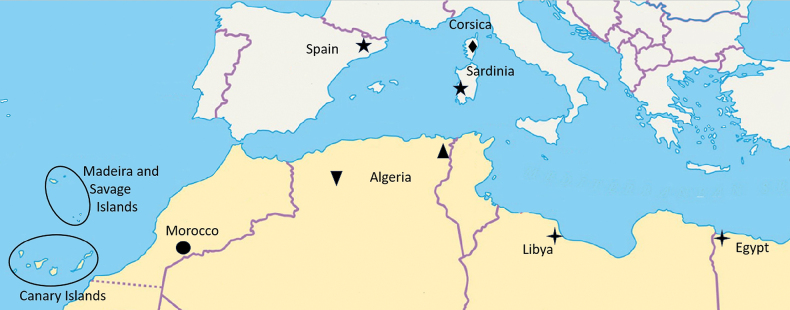
Location of known species of *Zimirina* from the Western Palearctic.

*Zimirina* taxa are known from very few endemic specimens, and descriptions are typically limited to one sex. This is the case for seven of the 13 species currently recorded in the Western Palearctic (Table 1, Fig. 17).

**Table 1.** Numbers of specimens listed for each of the known species of Palearctic *Zimirina*.

	♂	♀		♂	♀	Symbols
**Canary Islands**			**Morroco**			
*Z. cineris*	4	4	*Z. tenuidens*	1		●
*Z. gomerae*	1	1	**Algeria**			
*Z. grancanariensis*		1	*Z. deserticola*	1		▲
*Z. hirsuta*	6	2	*Z. penicillata*	2	2	▼
*Z. moyaensis*		2	**Libya, Egypt**			
*Z. nabavii*	1		*Z. vastitatis*		2	✦
*Z. spinicymbia*	1		**Spain, Sardinia**			
**Madeira and Savage Islands**			*Z. brevipes*		2	★
*Z. lepida*	18	34	**Corsica**			
			*Z. corsica*	1		♦

As a result, most of the descriptions are based on isolated and unpaired individuals. Consequently, intraspecific and intrapopulation variability is often unknown (Wunderlich 2011). The specimen found in Corsica could possibly be the male of the *Z. brevipes* females reported from Catalonia (Pérez and Blasco 1986) (Figs 20, 21) and also reported with some caution from Sardinia (Pantini et al. 2013) (Fig. 22). However, the association with the Sardinian female cannot be demonstrated with certainty. Depending on the families, the arachnofauna of Corsica can be very distinct from that of Sardinia, and the similarity between the two faunas remains low (Pantini et al. 2013). Considering the high level of endemism of species in this genus, the insular location of the specimen, and the unknown Corsican females, it is likely that the Corsican specimen belongs to a different species from the female specimens found in other Mediterranean areas. Without data on the presence of *Zimirina* males in Sardinia, and no barcoding data available at present, we hypothesize that the male Corsican specimen is a distinct species.

**Figures 18–22.**
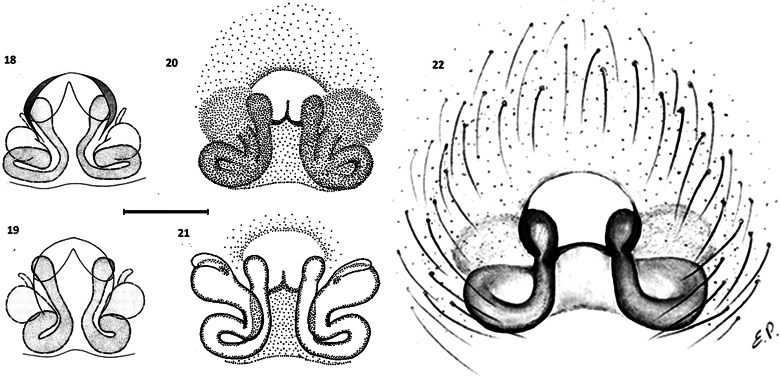
Epigynum of *Zimirina* females: **18, 19.** Cleared epigynum of two females of *Z. lepida* (according to Crespo et al. 2013); **20, 21.** Epigynum and vulva of *Z. brevipes* female from Spain (according to Pérez and Blasco 1986); **22.** Epigynum of *Z. brevipes* female from Sardinia (according to Pantini et al. 2013), although note that the epigynum shows notable differences between the two female specimens, such as the shape of the central opening as well as the height and width (scale line: 0.1mm).

## Supplementary Material

XML Treatment for Zimirina corsica

## ﻿Additional information

### Use of AI

No use of AI was reported.

### Author ORCIDs

Christine Rollard ￼ https://orcid.org/0009-0003-6922-845X

Olivier Villepoux ￼ https://orcid.org/0009-0005-5366-4681

Frédéric Ysnel ￼ https://orcid.org/0000-0003-0101-9005

### Data availability

All of the data that support the findings of this study are available in the main text.
